# Supplementary material for: Potent Activity of a High Concentration of Chemical Ozone against Antibiotic-Resistant Bacteria
Source: Molecules. 2022 Jun 22;27(13):3998. doi: 10.3390/molecules27133998 (PMC9268618; doi:10.3390/molecules27133998)
Supplement: Supplementary file 1 [file molecules-27-03998-s001.zip › molecules-1758239-supplementary.pdf]

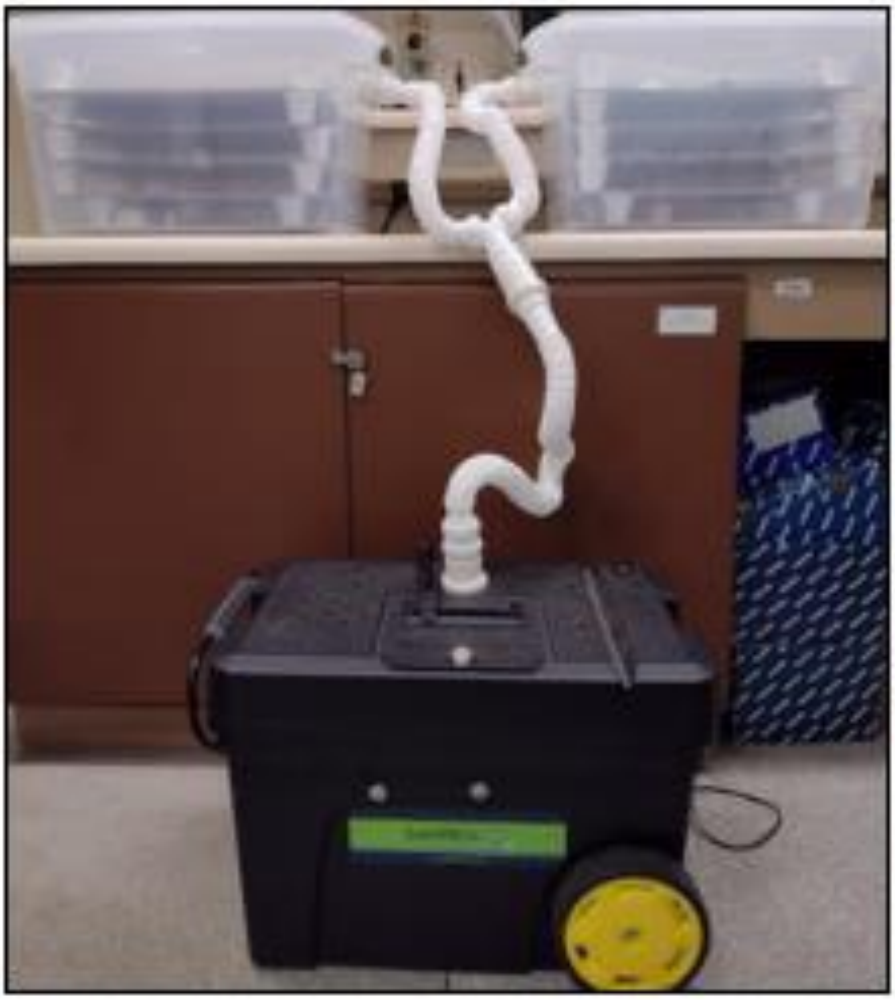

Figure S1: SANITECH O3-80-Sanitization equipment ozone generator coupled to two containers of approximately 1 m^3 each, used for exposing samples to ozone.
